# Report on the complete organelle genomes of *Orobanche Filicicola* Nakai ex Hyun, Y. S. Lim & H. C. Shin (Orobanchaceae): insights from comparison with Orobanchaceae plant genomes

**DOI:** 10.1186/s12864-025-11298-2

**Published:** 2025-02-17

**Authors:** Sang-Chul Kim, Eun Su Kang, Tae-Hee Kim, Ye-Rim Choi, Hyuk-Jin Kim

**Affiliations:** Division of Forest Biodiversity, Korea National Arboretum, 509 Gwangneungsumogwon-ro, Soheul-eup, Pocheon-si, 11186 Gyeonggi-do Republic of Korea

**Keywords:** Organelle genome, *Orobanche Filicicola*, Orobanchaceae, Phylogenomic

## Abstract

**Background:**

*Orobanche* is a parasitic plant distributed in the temperate zone of Northern Hemisphere, with approximately 200 species found worldwide. In the Republic of Korea, two species of *Orobanche*, namely *O. coerulescens* Stephan ex Willd. and *O. filicicola* Nakai ex Hyun, Y. S. Lim & H. C. Shin, are present, with *O. filicicola* being endemic. Genome analysis of this species has not yet been performed, and characterizing its complete organelle genome will provide valuable insights into the phylogeny and genome evolution of parasitic plants.

**Results:**

The chloroplast and mitochondrial genomes were analyzed, revealing distinct characteristics. The chloroplast genome is 91,529 bp long with a GC content of 33.6%, containing 33 protein-coding, 30 tRNA, and 4 rRNA genes. In contrast, the mitochondrial genome is 1,058,991 bp long with a GC content of 45.5%, featuring 31 protein-coding, 16 tRNA, and 3 rRNA genes. The mitochondrial genome has over three times more simple sequence repeats and longer long repeats than the chloroplast genome. Analysis of synonymous codon usage in protein-coding genes from nine Orobanchaceae species revealed significant differences between chloroplasts and mitochondria, with codons ending in A or T exhibiting higher coding rates. Ka/Ks ratio calculations indicated that *psbI* and *atpB* had the smallest and largest ratios in chloroplasts, respectively, while *ccmFC* was identified as the only gene under positive selection in mitochondria genomes. Sequence alignment identified 30 homologous fragments between the two genomes, totaling 7,247 bp. Comparison of *O. filicicola*’s chloroplast genome with related species showed gene loss and conserved inverted repeat sequences. Numerous homologous collinear blocks were found in mitochondrial genomes of related species, but some regions lacked homology. Phylogenetic analysis indicated identical topologies for chloroplasts and mitochondria, with Orobanchaceae forming a strong monophyletic group.

**Conclusions:**

Characterizing the complete organelle genome of *O. filicicola* enabled a comprehensive analysis of the Orobanchaceae organelle genome, providing important baseline data for its structure and evolution.

**Supplementary Information:**

The online version contains supplementary material available at 10.1186/s12864-025-11298-2.

## Background

Plant eukaryotic cells have chloroplasts and mitochondria, and both these organelles, originally independent organisms, have evolved through endocytosis and subsequent capture [1, 2]. Chloroplasts exist only in plant cells and have a double-layer membrane structure. The chloroplast genomes of higher plants are generally 115–165 kb in size and are highly conserved in structure [3]. In higher plants, the structure of tobacco chloroplasts was first identified [4]. Its genome consists of a circular DNA molecule with a typical structure consisting of four segments [two inverted repeat (IR) regions; IRa and IRb, and a large and a small single-copy regions; large single-copy (LSC) and small single-copy (SSC)]. In most plant chloroplast genomes, the LSC region is 81–90 kb, the SSC region is 18–20 kb, and the IR region varies in length from 5 to 76 kb [5, 6]. The LSC and SSC regions are separated by two IR sequences. The size of the chloroplast genome has changed by expansion of the IR, and inverted structures have been found mostly in the LSC region [7–14]. Recent studies have identified genomes that lack SSC or have only one IR [15, 16].

Mitochondria play a crucial role in energy synthesis and conversion for physiological activities of eukaryotes, thereby affecting plant growth and development [17, 18]. In particular, they produce ATP and are involved in cell division, differentiation, and apoptosis [19, 20]. According to the endosymbiotic theory, it originated from endosymbiosis of alpha bacteria and eventually evolved into a eukaryotic organelle, and like chloroplasts, it is maternally inherited, independently of the nucleus [21, 22]. Plant mitochondrial genomes considerably vary in size, from 60 kb to > 11 Mb, depending on the species, and their structures are circular, linear, or even complex branched and networked [23–27]. The mitochondrial genome is composed of a double-stranded circular DNA and has structural features more complex than that of the chloroplast genome owing to structural modifications caused by more repetitive sequences than those of the chloroplast genome; the number of genes encoded by the mitochondrial genome varies among plants; however, it has fewer genes than the chloroplast genome, and the types and sequences of genes are highly conserved. Of the three plant genomes, the mitochondrial genome evolves the slowest and most conservative in evolution [28–30].

Orobanchaceae Vent. consists of approximately 2,060 species spanning 90 genera distributed on all continents and major islands except Antarctica [31]. *Orobanche* species are non-chlorophyll annual or perennial plants, which parasitize the roots of various plant species. Approximately 200 *Orobanche* species are distributed in the temperate zone of Northern Hemisphere [32]. In Republic of Korea, two species, *O. coerulescens* Stephan ex Willd. and *O. filicicola* Nakai ex Hyun, Y. S. Lim & H. C. Shin, are predominant, with *O. filicicola* endemic to the region [33]. It is a parasitic plant that lives along riverbanks, and parasitically grows on *Artemisia* in gravel or sandy soil with little fallen leaves and good sunlight. The *Orobanche* species in Republic of Korea are threatened by habitat destruction, degradation, and fragmentation, and among the Korean species, *O. filicicola* is endangered [34]. Recently, a cytotaxonomy study of *Orobanche* has been conducted, and the bivalent chromosome and chromosome number have been confirmed (2n = 2x = 38) [35]. The species of Orobanchaceae have nuclear genome sizes ranging from 223 Mb to 10.7 Gb, and the closely related species *O. coerulescens* distributed in Republic of Korea has a genome of approximately 3.6 Gb [34, 36]. However, studies on the organelle genome of *O. filicicola* are scanty.

Therefore, in the present study, we determined the organelle genome of *O. filicicola* and compared it with previously reported genomes of Orobanchaceae for analyzing the size, gene content, intron content, and repeats of the parasitic plant organelle genome.

## Methods

### Plant sampling and DNA sequencing

*O. filicicola* was collected from Jeju Island (Republic of Korea), and vouchers (KHB1648819) were preserved in the herbarium of Korea National Arboretum. Genomic DNA was extracted from fresh stem tissue using a DNeasy Plant Mini Kit (Qiagen, Hilden, Germany). Paired-end libraries were constructed with an average insert size of 301 bp using Illumina Miseq (Illumina Inc., San Diego, CA, USA). Approximately 10 Gb paired-end reads were generated.

### Organellar genome assembly and annotation

Raw FASTQ reads were filtered using fastp v.0.23.4 [37] with default settings to filter out adapter sequences and low-quality reads. For chloroplast assembly, we utilized GetOrganelle v.1.7.7.1 [38] with the following parameters: ‘-R 30 -k 21, 55, 85, and 115 -F embplant_pt’ to assemble the Illumina reads. This resulted in generated two complete chloroplast genome sequences; however, they differed only in the orientation of the SSC region. Therefore, we selected the one with the SSC region aligned in the orientation same as that in *Rehmannia chingii* H. L. Li (OR601178). We used ‘map to reference’ in Geneious Prime v.2024.0.7 to distinguish between used and unused reads to assemble the chloroplast genome [39]. For mitochondrial assembly, unused reads in the chloroplast genome assembly were assembled into a mitochondrial scaffold using GetOrganelle with parameters ‘-R 100 -k 21, 55, 85, 115 -F embplant_mt, -P 100000 --memory-save’. The assembled scaffold was expanded and reassembled using Unicycler v.0.5.0 [40] and Geneious Prime to finally complete the three chromosomes. To validate the assembly results, we used BWA [41] to map all reads to the completed organelle genome and visualized them using the ‘Advanced circos’ module in TBtools-II v.2.119 [42] to determine the coverage depth.

The *O. filicicola* chloroplast genome was annotated using Geseq [43]. The mitochondrial genome was initially annotated using Geneious Prime by referencing the mitochondrial genomes of *Osmanthus fragrans* Lour. (MW645067) [44], *Rehmannia glutinosa* (Gaertn.) DC. (OM397952) [45], and *Salvia miltiorrhiza* Bunge (KF177345). The genome was then finalised using PMGA for complete annotation [46]. All tRNA genes were predicted using tRNAscan-SE v.1.3.1 [47], and the chloroplast genome and mitochondrial genome maps were generated using OrganellarGenomeDRAW (OGDRAW) [48].

### Repeat sequence detection

Using REPuter online program [49], repeated sequences were detected in the chloroplast genome by setting Hamming distance to 3 (sequence consistency ≥ 90%), and repeated sequences in the mitochondrial genome were detected by setting the minimum repeat size to 30 bp.

Simple sequence repeats (SSRs) were detected using Krait v.1.5.1 [50] with the following settings: the minimum repeat unit numbers for mononucleotides, dinucleotides, and trinucleotides were 10, 5, and 4, respectively, and the minimum repeat unit numbers for tetranucleotides, pentanucleotides, and hexanucleotides were 3.

### Codon usage bias and Ka/Ks analysis

For the analysis of relative synonymous codon usage (RSCU) in organelle genomes, protein coding genes from chloroplasts and mitochondria were extracted from Geneious Prime. RSCU and codon frequency analyses were performed using DnaSP v.6.12.03 [51]. Codons with RSCU values ​​> 1 were defined as optimal codons.

To determine the Ka/Ks ratio, nonsynonymous (Ka) and synonymous (Ks) mutations were determined in DnaSP using *Salvia miltiorrhiza* Bunge as a reference.

### Identification of mitochondrial chloroplast DNA [mitochondrial-to-chloroplast DNA transfer (MTPT)]

BLASTN v.2.13.0 [52] was used to discriminate mitochondrial chloroplast DNA (MTPT) between mitochondrial genomes and chloroplast genomes (minimum identity 80%, e-value cutoff 1 × 10^− 5^). Additionally, repeated fragments were uniquely marked to ensure accurate detection. Results were visualized in ‘Advanced circos’ module of TBtools-II.

### Comparison of organelle genomes

First, the chloroplast genomes were aligned using MAFFT [53]. Complete chloroplast genomes of nine species were compared using mVISTA [54], with *R. chingii* as a reference.

Mitochondrial genomes were compared by synteny analysis. BLASTN was used to compare mitochondrial genomes with each other. Then, homologous sequences with a length ≥ 500 bp were extracted, and multi-Chr layouts, gene links, and.gff files among Orobanchaceae were generated by One Step MCScanX-Super Fast module of TBtools-II, with an E-value of 1 × 10^− 6^. Homologous genes between other species were obtained from the merged gene link file after merging files of the comparison groups using ‘Text Merge for MCScan-X’ module. Collinearity plots between species were visualized by ‘Multiple Synteny Plot’ module of TBtools-II.

### Organellar phylogenetic inference

We downloaded the sequences of species containing both mitochondrial and chloroplast genomes of Orobanchaceae and their close relatives from the National Center for Biotechnology Information (NCBI) database [55]. Additionally, we manually annotated and corrected the annotation errors in these sequences. We extracted protein-coding genes (PCGs) of chloroplasts and mitochondria using Geneious Prime. We selected the optimal model with ModelFinder v.1.6.8 [56] in Phylosuit v.1.2.2 [57] and performed maximum likelihood (ML) analysis with 1000 bootstrap (BS) iterations in IQ-Tree2 [58, 59]. The final phylogenetic tree was visualized in Figtree v.1.4 (http://tree.bio.ed.ac.uk/software/figtree).

## Results

### Genome assembly and characterization

**Fig. 1:**
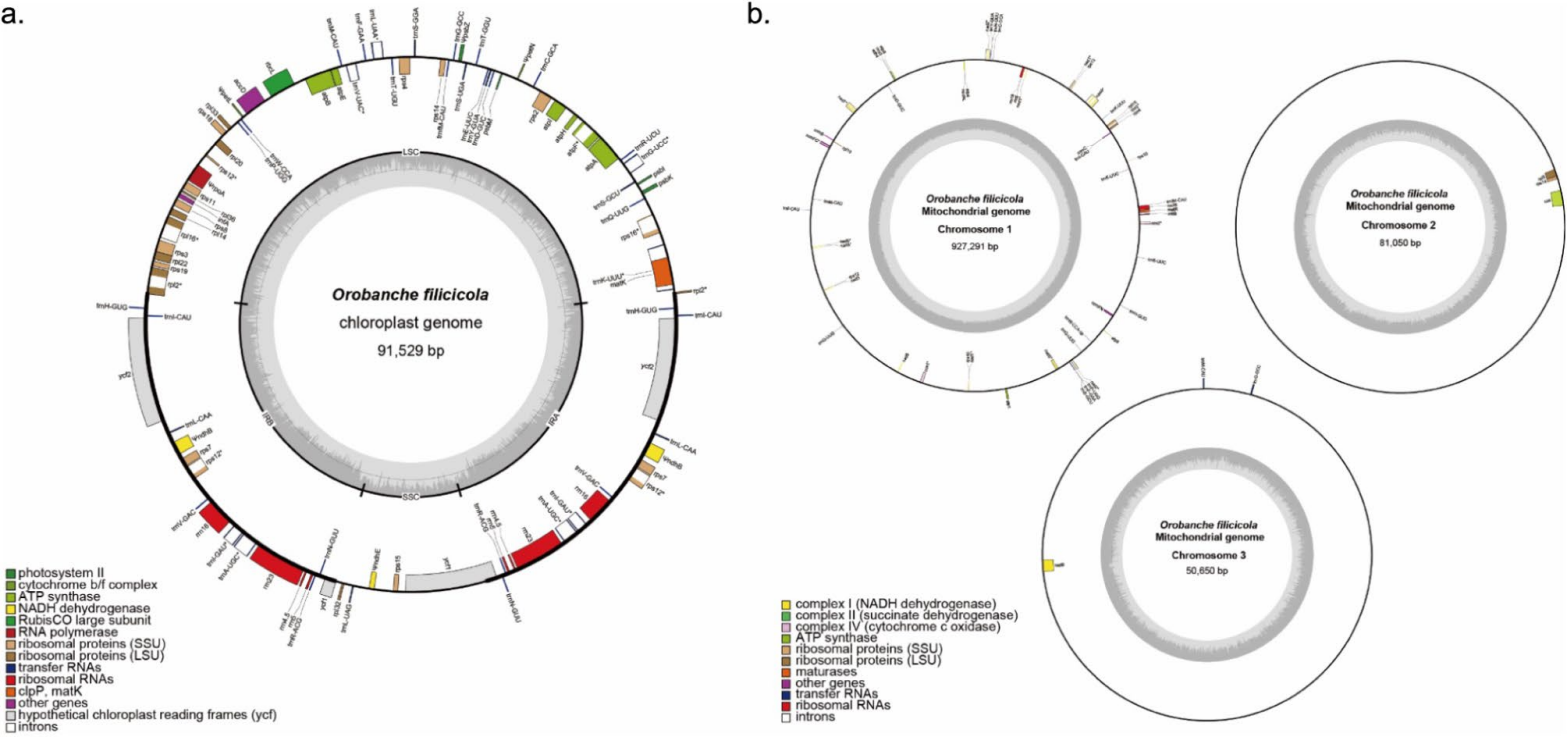
Organelle genome diagrams of *O. filicicola*. **(a)** Chloroplast genome diagram. **(b)** Mitochondrial genome diagram

We combined the sequencing data from the Illumina platform and successfully assembled the accurate organelle genomes of *O. filicicola*. After filtering, we obtained a total of 34,536,866 clean reads, totaling 10,224,733,148 bp. The chloroplast genome (Fig. 1a) had 151,236 reads, totaling 91,529 bp (average depth: 919.78 x; Fig. S1), and the mitochondrial genome (Fig. 1b) had 219,470 reads, totaling 1,058,991 bp with three chromosomes (Table 1). The assembled chloroplast genome of *O. filicicola* had a total length of 91,529 bp and contained 84 genes, comprising 38 protein-coding, 38 tRNA, and eight rRNA genes; the GC content was 33.6% (Table 2). The *O. filicicola* mitochondrial genome formed three circular chromosomes. Chromosome 1 was 927,291 bp (average depth: 130.89 x; Fig. S2) with a GC content of 45.1% (accession number: PQ467906); chromosome 2 was 81,050 bp (average depth: 135.26 x; Fig. S2) with a GC content of 44.7% (PQ467907); and chromosome 3 was 50,650 bp (average depth: 123.25 x; Fig. S2) with a GC content of 45% (PQ467908), and the total length was 1,058,991 bp. The *O. filicicola* mitochondrial genome contained 53 genes, comprising 31 protein-coding, 19 tRNA, and three rRNA genes. The protein coding region of the *O. filicicola* mitochondrial genome was 28,419 bp in length, with 42.5% GC content. The genes *rps3*,* cox1*,* cox2*, and *ccmFC* contained one intron each. The genes *nad4* and *nad7* contained three introns each and *nad1*, *nad2* and *nad5* contained four introns each (Table 3).

**Table 1:** Information on Illumina Miseq data and organelle data of *O. Filicicola*

	Total bases (bp)	Total reads	GC (%)	AT (%)
Raw file	10,893,648,724	36,191,524	41	59
file filtered by fastp	10,224,733,148	34,536,866	40.8	59.2
Files assembled into the chloroplast	91,529	151,236	33.6	66.4
Files assembled into the mitochondria	1,058,991	219,470	45.5	54.5
Chromosome 1	927,291	191,379	45.1	54.9
Chromosome 2	81,050	18,181	44.7	55.3
Chromosome 3	50,650	9,910	45	55

**Table 2:** Gene content and functional classification of the chloroplast genome in *O. Filicicola*

Category for genes	Group of genes	Genes
Self-replication	Large subunit ribosomal proteins	rpl2*,* rpl14*, rpl16*,* rpl20*,* rpl22*,* rpl32*,* rpl33*,* rpl36*
	DNA-dependent RNA polymerase	Ψ*rpoA*
	Small subunit ribosomal proteins	*rps2*,* rps3*,* rps4*,* rps7*(x2), *rps8*,* rps11*,* rps12*(x2)*,* rps14*,* rps15*, rps16*,* rps18*,* rps19*
	Ribosomal RNAs	*rrn4.5 S*(x2), *rrn5S*(x2), *rrn16S*(x2), *rrn23S*(x2)
	Transfer RNAs	*trnA-UGC*(x2)*,* trnC-GCA*,* trnD-GUC*,* trnE-UUC*,* trnF-GAA*,* trnfM-CAU*,* trnG-GCC*, trnG-UCC*,* trnH-GUG*(x2), *trnI-GAU*(x2)*,* trnI-CAU*(x2), trnK-UUU*,* trnL-CAA*(x2), trnL-UAA*,* trnL-UAG*,* trnM-CAU*,* trnN-GUU*(x2), *trnP-UGG*,* trnQ-UUG*,* trnR-ACG*(x2), *trnR-UCU*,* trnS-GCU*,* trnS-GGA*,* trnS-UGA*,* trnT-GGU*,* trnT-UGU*,* trnV-GAC*(x2), trnV-UAC*,* trnW-CCA*,* trnY-GUA*
Photosynthesis	Subunits of ATP synthase	*atpA*,* atpB*,* atpE*, atpF*,* atpH*,* atpI*
	Subunits of NADH dehydrogenase	*ΨndhB*(x2)*,* ΨndhE*
	Subunits of cytochrome b/f complex	*ΨpetL*,* ΨpetN*
	Subunits of photosystem I	*ΨpsaC*
	Subunits of photosystem II	*psbI*,* psbK*,* psbM*,* ΨpsbZ*
	Subunit of rubisco	*rbcL*
Other genes	Subunit of acetyl-CoA-carboxylase	*accD*
	Translational initiation factor	*infA*
	maturase	*matK*
Unknown function	Conserved open reading frames	*ycf1*,* ycf2*(x2)

Note: (×2), two gene copies in IRs; *, gene containing a single intron; **, gene containing two introns; Ψ, pseudogene

**Table 3:** Gene content and functional classification of the mitochondrial genome in *O. Filicicola*

Group of genes		Genes
Core genes	Complex I (NADH dehydrogenase)	nad1^****^, nad2^****^, *nad3*, nad4^***^, *nad4L*, nad5^****^, *nad6*, nad7^***^, *nad9*
	Complex III (ubiquinol-cytochrome c reductase)	*cob*
	Complex IV (cytochrome c oxidase)	cox1^*^, cox2^*^, *cox3*
	ATP synthase	*atp1*,* atp4*,* atp6*,* atp8*,* atp9*
	Cytochrome c biogenesis	*ccmB*,* ccmC*, ccmFC^*^, *ccmFN*
	Maturases	*matR*
	Protein transport subunit	*mttB*
Variable genes	Complex II (succinate dehydrogenase)	*Ψsdh4*
	Ribosomal protein large subunit	*rpl5*,* rpl10*,* rpl16*
	Ribosomal protein small subunit	rps3^*^, *rps4*,* rps12*,* rps14*
rRNA genes	Ribosome RNA	*rrn5*,* rrn18*,* rrn26*
tRNA genes	Transfer RNA	*trnC-GCA*,* trnD-GUC*,* trnE-UUC*(x2), *trnF-GAA*,* trnfM-CAU*,* trnG-GCC*,* trnH-GUG*,* trnI-CAU*,* trnK-UUU*,* trnL-CAA*,* trnM-CAU*(x2), *trnN-GUU*,* trnP-UGG*,* trnQ-UUG*(x2), *trnW-CCA*,* trnY-GUA*

Note: *, number of introns included in the gene; (×2), two gene copies; Ψ, pseudogene

### Repeat sequence analysis of organelle genomes

The distribution of SSRs was analyzed in nine Orobanchaceae chloroplast genomes using Krait. The lowest number of repeats (18) was identified in *Orobanche cernua* var. *cumana* (Wallroth) Beck, while the highest number (83) was identified in *Christisonia kwangtungensis* (Hu) G.D.Tang, J.F.Liu & W.B.Yu. Mononucleotide repeats were the most abundant in all these species. The second most abundant repeat sequences were mostly composed of dinucleotide repeats, with tetranucleotides identified in *R. glutinosa* and *R. chingii*. Most SSRs contained A/T motifs. *O. filicicola*, assembled in this study, had 50 SSRs, making it the fourth most abundant among the analyzed species (Fig. S3; Table S1). In mitochondria, the lowest number of repeats (56) was identified in *Pedicularis kansuensis* Maxim., while the highest number of repeats (183) was identified in *O. filicicola*. All species had tetranucleotide repeats as their most abundant sequence. The second most abundant repeat sequence was composed of dinucleotide repeats, which were identified as trinucleotides in the case of *Cistanche deserticola* Y. C. Ma (Fig. S4; Table S2).Long-repeat analysis revealed that more forward and palindromic repeats were identified than reverse and complementary repeats in the nine Orobanchaceae chloroplast genomes. Complement repeats have been identified in *Ch. kwangtungensis* (1), *Castilleja paramensis* F. González&Pabón-Mora (5), and *R. glutinosa* (3). Repeat sizes of 20 or less were identified as 1–35, repeat sizes of 21–30 were identified as 11–37, repeat sizes of 31–40 were identified as 1–10, repeat sizes of 41–50 were identified as 1–8, and repeat sizes of 51–60 were identified as 1 each in species except *O. cernua* var. *cumana*, *Ca. paramensis*, and *P. kansuensis*, while repeat sizes of 100 or more were identified only in *Ch. kwangtungensis* (Fig. S5; Table S3). For mitochondria, only forward and palindromic repeats were identified. The number of repeats ranged from a minimum of 27 in *O. cernua* var. *cumana* to a maximum of 61 in *C. deserticola*. Specifically, the repeat size of 30 was recorded as ranging from 1 to 9, while the repeat size of 31 to 40 ranged from 9 to 31. For repeat sizes of 41 to 50, the range was 1 to 17, and for sizes of 51 to 60, it was 1 to 13. Notably, repeat sizes of 401 to 500 were identified only in *Aeginetia indica* L. and *Ca. paramensis*, whereas repeat sizes of 601 to 700 were exclusively found in *O. filicicola* (Fig. S6; Table S4).

### Codon usage and Ka/Ks analysis of protein coding genes

**Fig. 2:**
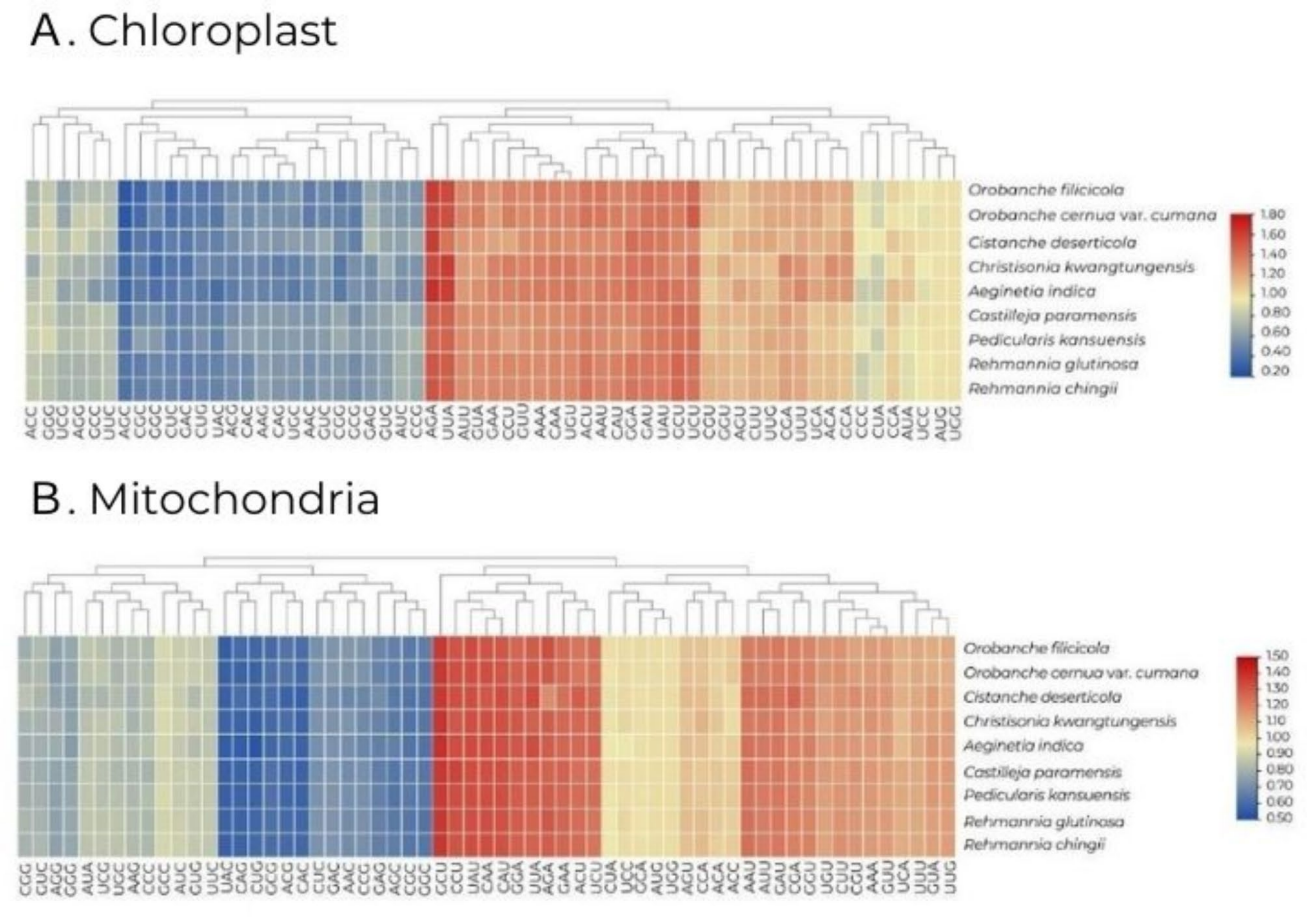
The RSCU values of nine Orobanchaceae organelle genomes. **(A)** The RSCU values of nine Orobanchaceae chloroplast genomes. **(B)** The RSCU values of nine Orobanchaceae mitochondria genomes

The relative frequency of synonymous codon usage, excluding three stop codons, was estimated using the PCGs of the organelles from nine species of the Orobanchaceae family. For chloroplasts, the number of codons ranged from 5,114 in *A. indica* to 26,165 in *R. chingii*. A heat map was generated based on the RSCU results (Fig. 2A). The evolutionary tree was divided into two major branches according to the RSCU values of 61 codons. The first branch comprised 35 codons with RSCU values greater than 0.72, while the second branch included the remaining codons. High similarity in codon usage was observed among the nine Orobanchaceae species. Codons ending with A or T exhibited higher coding rates. With the exception of tryptophan (UGG), serine (UCC), and proline (CCC), codons ending with A or T had RSCU values greater than 1, whereas those ending with C or G had RSCU values less than 1 (Table S5). For mitochondria, the number of codons ranged from 7,215 in *C. deserticola* to 8,532 in *Ch. kwangtungensis*, and a heat map (Fig. 2B) was generated based on the RSCU results. Similar to chloroplasts, the evolutionary tree was divided into two major branches based on the RSCU values of 61 codons. The first branch consisted of 34 codons with RSCU values greater than 0.90, while the second branch included the remaining codons. High similarity in codon usage was also noted among these nine Orobanchaceae species. Codons ending with A or T showed higher coding rates, and except for tryptophan (UGG) and threonine (ACC), codons ending with A or T had RSCU values greater than 1, whereas those ending with C or G had RSCU values less than 1. Although codons in organelles vary slightly, amino acids typically possess at least two synonymous codons, with arginine (Arg), leucine (Leu), and serine (Ser) being the most abundant, each having six codons. Both methionine (AUG) and tryptophan (UGG) exhibited RSCU values of 1 (Table S6).

**Fig. 3:**
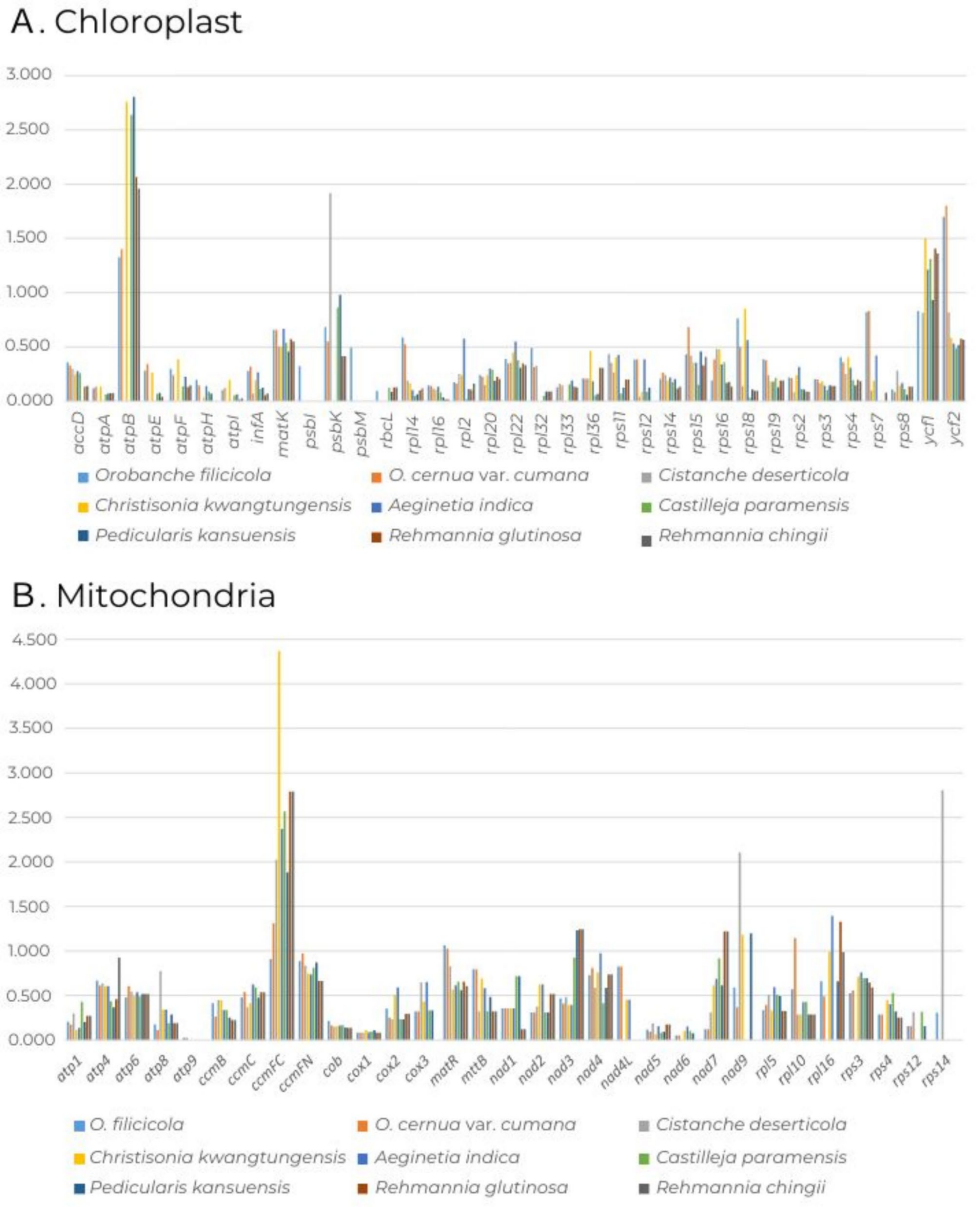
The Ka/Ks ratios of CDSs from nine Orobanchaceae organelle genomes for comparison with *Salvia miltiorrhiza*. Ka/Ks ratios > 1 indicate strong positive selection. **(A)** Ka/Ks ratios of 35 CDSs from the chloroplast genome. **(B)** Ka/Ks ratios of 31 CDSs from the mitochondrial genome

The Ka/Ks ratios were calculated for 35 protein-coding genes based on the chloroplast genome of *O. filicicola* assembled in this study (Fig. 3A). Although variations were observed due to differing numbers of chloroplast genes among species, the genes with the smallest and largest average Ka/Ks ratios were *psbI* (0.085) and *atpB* (2.136), respectively. The genes inferred to have undergone positive selection included *atpB* and *ycf1*, both exhibiting average Ka/Ks ratios greater than 1 (Table S7). For mitochondria, the ratios were calculated for 31 protein-coding genes. The smallest average ratio was observed for *atp9* (0.005), while the highest ratio was found in *ccmFC* (2.335). Notably, *ccmFC* was the only gene inferred to have undergone positive selection (Fig. 3B; Table S8).

### Characteristics of mitochondrial-chloroplast DNA sequences (MTPTs)

**Fig. 4:**
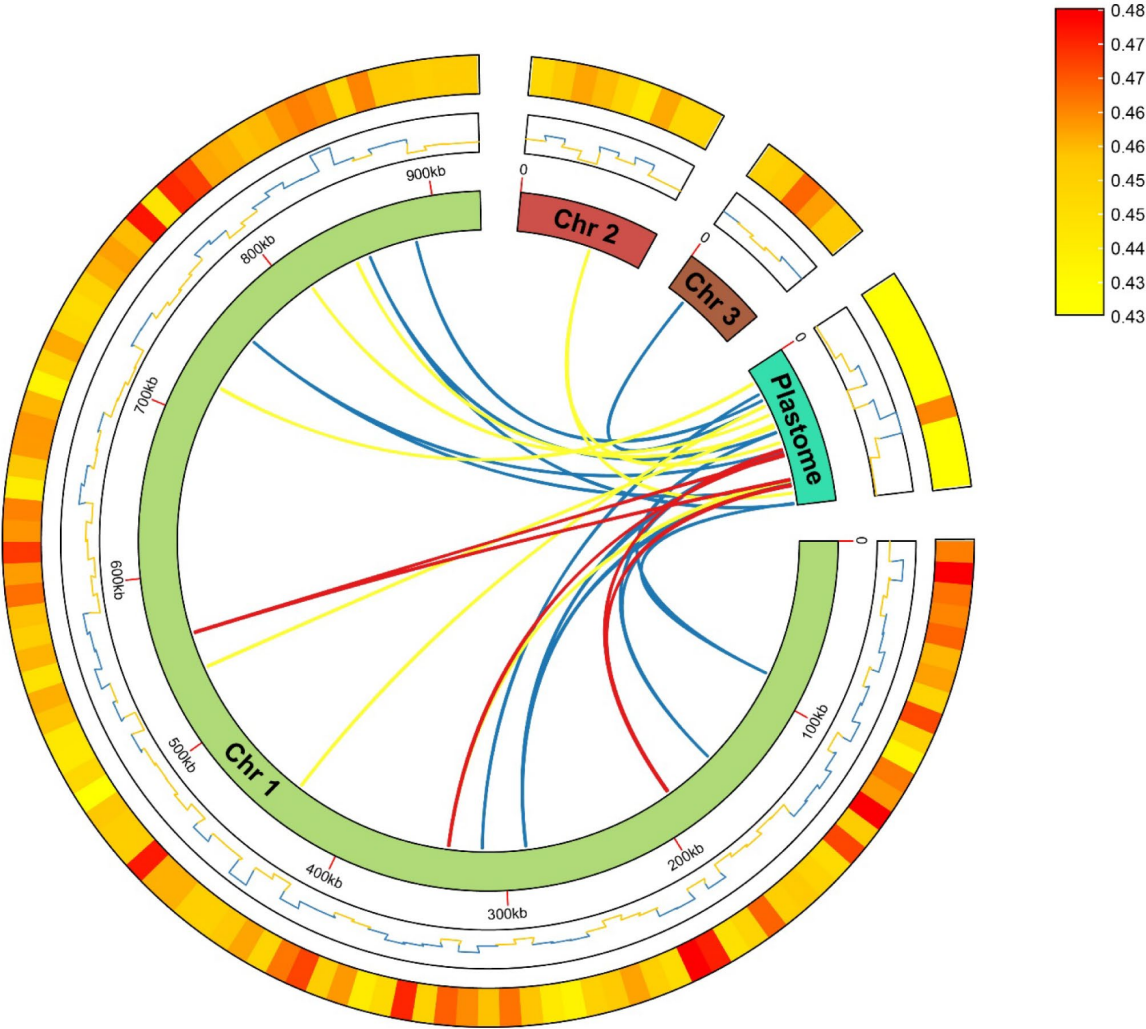
Gene transfer between the chloroplast and mitochondrial genomes of *O. filicicola*. The four arcs represent three mitochondrial chromosomes and the chloroplast genome, respectively, and lines connecting the arcs represent homologous genome segments transferred between the two organelles (red lines, ≥ 500 bp; blue lines, 150–500 bp; yellow, ≤ 150 bp). The middle line represents GCskew, and the last heatmap represents GC content

The mitochondrial genomes of higher plants often undergo extensive sequence transfers from chloroplast and even nuclear genomes. In our study, we annotated the chloroplast genome and compared it with the mitochondrial genome. Using BLASTN, we identified 30 MTPTs between the two organelle genomes. The combined length of these 30 MTPTs was 7,247 bp, accounting for 7.92% of the entire chloroplast genome and 0.68% of the mitochondrial genome. The maximum length was 889 bp, while the minimum length was only 31 bp. Five tRNA genes, such as *trnD-GUC*, *trnI-CAU*, *trnH-GUG*, *trnM-CAU*, and *trnW-CCA*, had transferred. Additionally, we identified several gene fragments in the chloroplast movement, including *rrn23*, *rrn16*, *rpl16*, *rps3*, and *atpA* (Table S9). These gene fragments may have undergone sequence loss during the movement process. A schematic of MTPT is provided in Fig. 4.

### Comparison of synteny analyses between chloroplast and mitochondrial genomes

**Fig. 5:**
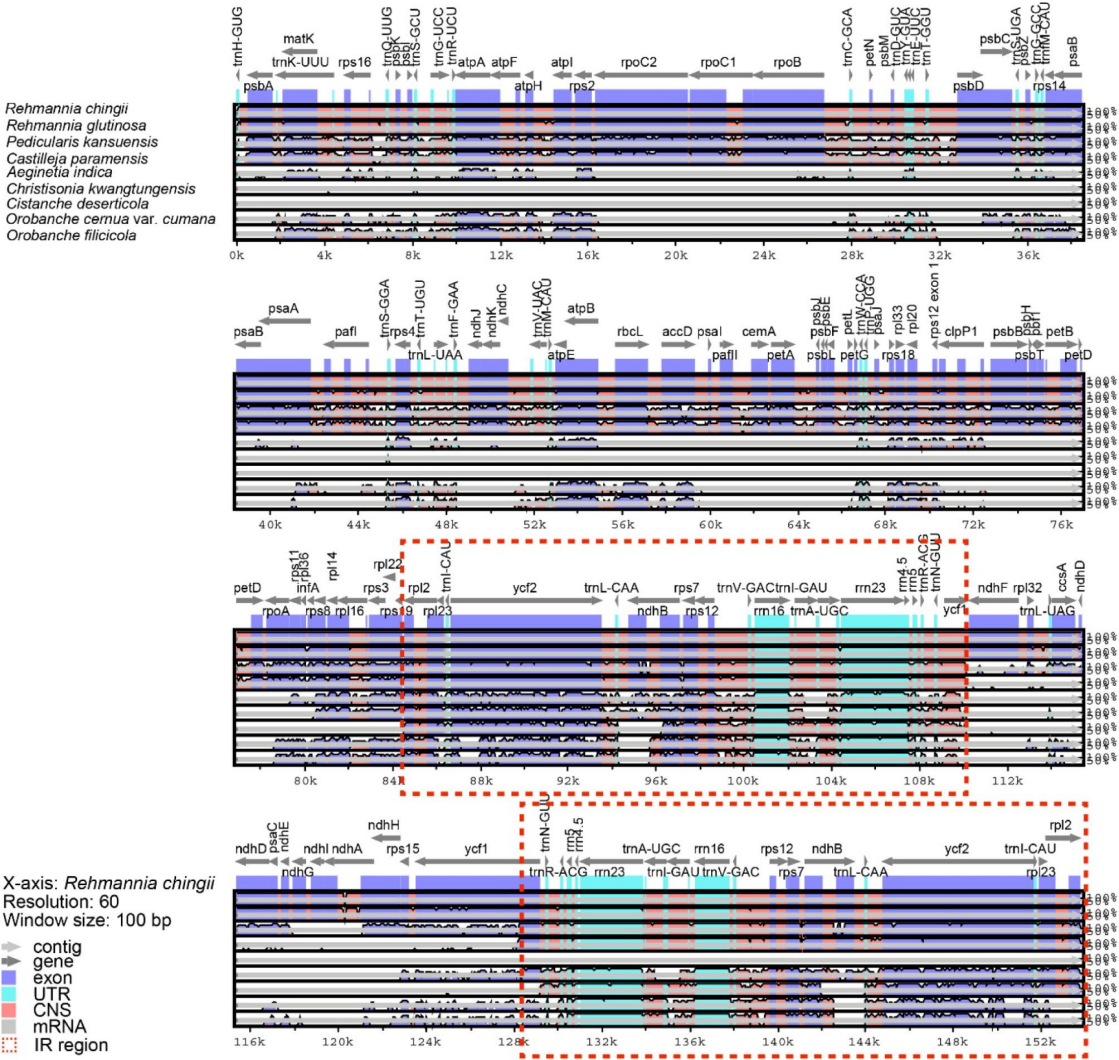
Comparison of the chloroplast genomes among nine Orobanchaceae species using m-VISTA. Gray arrows and thick black lines above the alignment indicate gene orientation. Purple bars indicate exons; blue bars indicate RNA; pink bars indicate noncoding sequences; gray bars indicate mRNA; and white peaks indicate differences in gene sequence. The y-axis indicates percent identity (range, 50–100%)

**Fig. 6:**
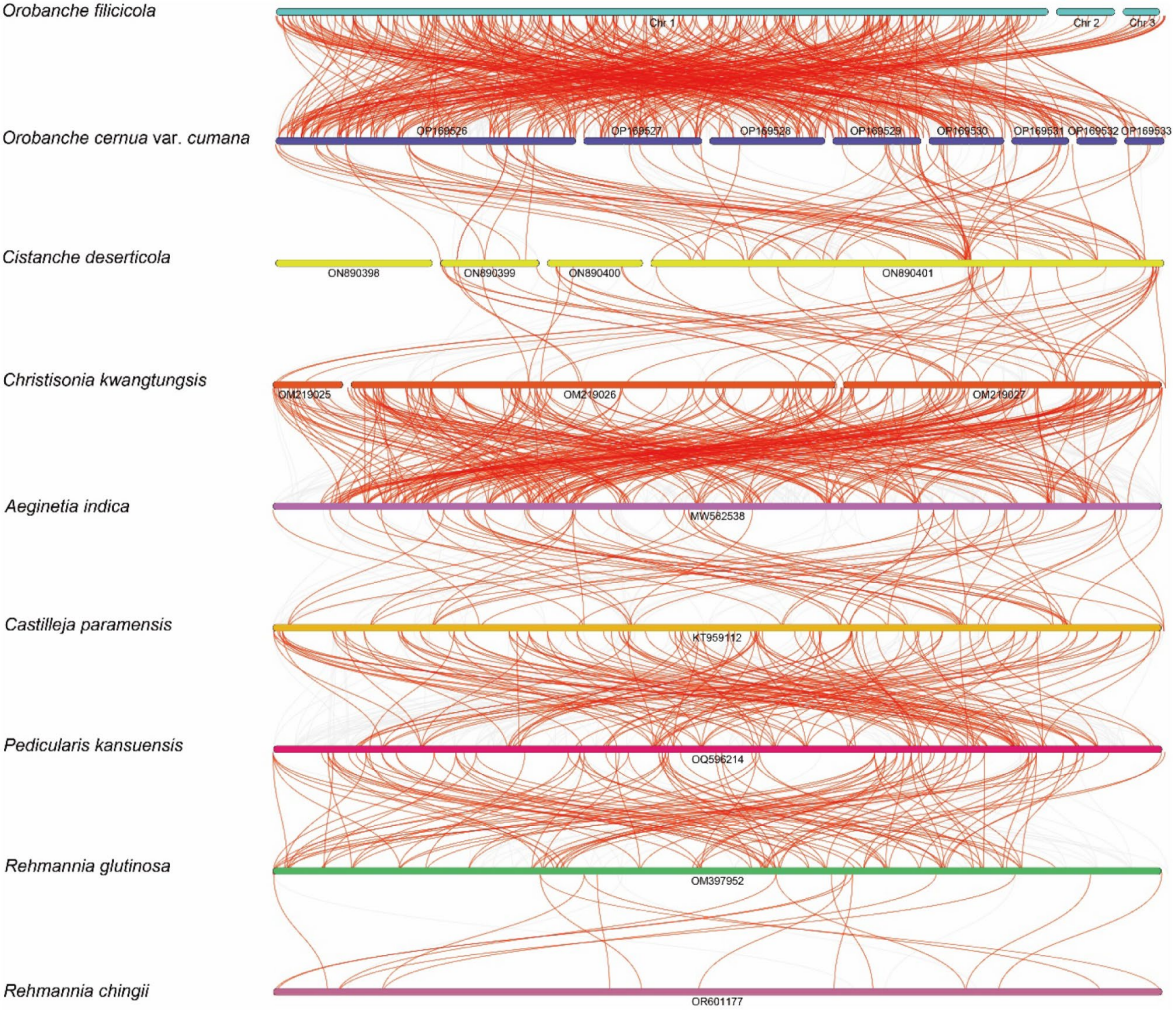
Multiple Synteny plots of the mitochondrial genomes of nine Orobanchaceae species. The bars on the graph indicate the mitochondrial genomes, while the ribbons depict the homologous sequences between adjacent species. The red areas highlight the positions of inversions, and the gray areas indicate regions with strong homology

To explore rearrangements and conserved sequence blocks within the chloroplast and mitochondrial genomes, we used mVISTA and BLASTN programs to identify homologous collinear blocks. Comparison of the chloroplast gene sequences and gene contents between nine Orobanchaceae species using mVISTA revealed significant differences in gene contents owing to gene loss in some species. Coding regions were more conserved than noncoding regions, and IR regions were more conserved than LSC and SSC regions (Fig. 5). In case of mitochondria, each ribbon connecting the mitochondrial genomes of nine Orobanchaceae species represents a highly homologous collinear block or sequence. The analysis showed the presence of numerous homologous collinear blocks. The longest collinear block was identified between *O. filicicola* and *O. cernua* var. *cumana*, and its length was 11,181 bp. The arrangement of the collinear blocks varied across individual mitochondrial genomes, suggesting that the mitochondrial genome of *O. filicicola* had undergone extensive genome rearrangements and had a highly unconserved structure compared to those of closely related species (Fig. 6).

### Phylogenetic analysis

**Fig. 7:**
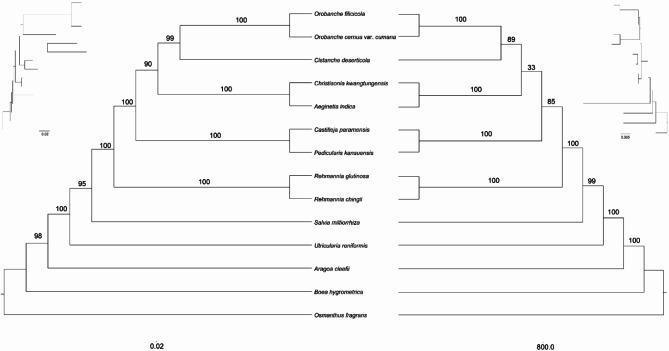
Comparison of ML phylogenetic tree based on are 34 protein-coding genes from the chloroplast genomes of 14 Lamiales species (left) and ML phylogenetic tree based on 31 protein-coding genes from the mitochondrial genomes of 14 Lamiales species (right). The numbers above the lines indicate ML posterior probabilities

We performed phylogenetic analyses using chloroplast and mitochondrial genomes of 14 angiosperm species, with five species of Lamiales as outgroups. The list of species used in the analyses and their corresponding GenBank accessions can be found in Table S10. We aligned and concatenated 35 shared PCGs for chloroplasts and 31 shared PCGs for mitochondria to generate matrices. Phylogenetic analyses yielded ML trees with strong support along the major basal branches (Fig. 7). Orobanchaceae formed a strong monophyletic group. *Rehmannia* species (*R. chingii* and *R. glutinosa*) clustered in the basal subclade with bootstrap values ​​of 100%, followed by *P. kansuensis* and *Ca. paramensis*, which formed a monophyletic group (BS = 100%). *Aeginetia indica* formed a strong monophyletic group with *Ch. kwangtungsis* (BS = 100%). The studied taxon, *O. filicicola*, was most closely related to *O. cernua* var. *cumana*. The mitochondrial genomes and chloroplast genomes trees of all 14 Lamiales species showed *O. fragrans* (Oleaceae) as the basal taxon, followed by *Boea hygrometricum* (Gesneriaceae), *Aragoa cleefii* (Plantaceae), *Utricularia reniformis* (Lentibuliaceae), and *Salvia miltiorrhiza* (Lamiaceae).

## Discussion

Third-generation sequencing platforms are widely used to analyze the genomes of plants with many repetitive sequences because they obtain longer base sequences than do second-generation sequencing platforms [60–62]. However, the methods are error-prone and suffer from the disadvantages of requiring DNA at high concentrations when analyzed using only third-generation sequencing. We had difficulty extracting high-concentration DNA from *O. filicicola*, because it has no leaves and is small in size compared to other angiosperms. To address this issue, we obtained a large amount of data from a second-generation sequencing platform and analyzed them and constructed complete organelle genome. The size of the chloroplast genome was 91,529 bp, similar to the size of other parasitic plants, and the loss of photosynthetic genes was noticeable (Fig. 1a). PCGs accounted for approximately 38.43% of the chloroplast genome; tRNA and rRNA genes accounted for 3.12% and 9.87%, respectively; and the remainder was noncoding sequences, accounting for 48.58%. In contrast, the mitochondrial genome consisted of three complete circular chromosomes arranged in order of size. The size was relatively large, showing chromosome 1 with 927,991 bp, chromosome 2 with 81,050 bp, and chromosome 3 with 50,650 bp, constituting a total of 1,058,991 bp. PCGs accounted for only approximately 2.61% of the mitochondrial genome; tRNA and rRNA genes accounted for 0.12% and 0.52%, respectively; and the remainder was identified as noncoding sequences, accounting for 96.75%. Our analysis results confirm that the mitochondrial genome has not a single circular structure but a circular multi-structure, which has been confirmed in previous studies using plant mitochondria and is generally owing to repetitive sequences [18, 20, 28, 63–65]. In addition, repetitive sequences significantly contribute to the size of mitochondria. As previously observed, the mitochondrial genome was larger than the chloroplast genome; however, it had relatively few genes [20, 45, 46, 63]: the chloroplast genome contained 84 genes, while the mitochondrial genome contained 53 genes. In *O. filicicola*, the chloroplast genome had 50 SSRs and 49 long repeat sequences, while the mitochondrial genome had 183 SSRs and 50 long repeat sequences, and the SSRs showed more than 3-fold difference, confirming that they significantly contributed to the size and structure. The number of long repeat sequences did not significantly differ; however, the lengths were considerably different. The maximum length of the chloroplast genome was 99 bp, while that of the mitochondrial genome was 1,071 bp.

Eukaryotic genomes contain 20 different amino acids and 61 codons (excluding three stop codons). Except for methionine and tryptophan, all amino acids are encoded by two to six synonymous codons. The analysis of codon preference and individual amino acid codon usage for PCGs from the two organelles of nine Orobanchaceae species is presented in Fig. 2. The PCGs in these organelles typically begin with the ATG start codon and preferentially end with A or U in the stop codons. These findings are consistent with previously reported codon preferences [18, 20, 24, 27, 45, 46, 56, 61, 63]. The chloroplast genome contains 29 codons with high codon usage (RSCU > 1), all identified as A/U. In contrast, there are 30 codons with low codon usage (RSCU < 1), predominantly identified as C/G. For mitochondria, 30 codons exhibited high codon usage, while 29 codons were identified with low codon usage. In the case of chloroplasts, a significant difference in the number of codons was observed between species when compared to mitochondria. This discrepancy appears to be attributed to the variation in the number of protein-coding genes within chloroplasts among species. Most Orobanchaceae species are parasitic plants, which suggests that this phenomenon is likely a result of the reduction in chloroplast size.

The Ka/Ks analysis serves as an important tool in molecular evolutionary studies to evaluate selective pressure on gene sequences within organelle genomes. Compared to Ka substitutions, Ks substitutions are generally more frequent across most organismal genes. Therefore, Ka/Ks values are typically less than 1. A Ka/Ks ratio greater than 1 indicates positive selection, whereas a ratio less than 1 suggests purifying selection. In this study, most protein-coding genes exhibited Ka/Ks ratios < 1, indicating purifying selection. Notably, two genes in the chloroplast (*atpB* and *ycf1*) and one gene in the mitochondria (*ccmFC*) were identified as exhibiting strong positive selection (Fig. 3).

Intracellular gene transfer is the transfer of sequences between the genomes of two organelles, particularly from the mitochondrial to the chloroplast genome. Our study identified 30 fragments of homologous sequences between the chloroplast and mitochondrial genomes, totaling 7,247 bp, including eight complete tRNA genes. This phenomenon is common in angiosperms, demonstrating the existence of gene transfer between chloroplasts and mitochondria [18, 20, 28, 56, 61–63].

Comparative analysis of the organelle genomes of nine Orobanchaceae species revealed that chloroplast genomes are more conserved in coding regions than in noncoding regions, and more conserved in IR regions than in LSC and SSC regions. However, parasitic species showed clear differences due to the loss of certain genes. Collinearity analysis of mitochondrial genomes showed that the mitochondrial genome of *O. filicicola* has undergone extensive rearrangements, and that phylogenetically closer species have more collinear regions. Given the limitations of our current study, future investigations should incorporate third-generation sequencing and advanced techniques, such as Hi-C or Pore-C, to more comprehensively explore three-dimensional genome architecture.

While chloroplast genomes have been widely used in phylogenetic studies, mitochondrial genomes have been less commonly utilized. The phylogenetic results of this study based on the mitochondrial genome were consistent with those based on the chloroplast genome and with previous studies [56]. These results suggest that some conserved gene clusters in plant mitochondrial genomes can be used as signals for phylogenetic analysis. Our findings may have implications for future research on the genetics, growth, and development of *O. filicicola*, an endemic and endangered species in the Republic of Korea.

## Conclusions

Comprehensive analysis of organelle genomes of Orobanchaceae has improved our understanding of their structure and evolution. Identification of SSRs and homologous blocks in the organelle genomes of *O. filicicola* and related species opens avenues for genetic improvement and restoration studies of endangered species. Overall, this study highlights the importance of understanding organelle genome structures in the areas of basic and applied plant science.

## Electronic supplementary material

Below is the link to the electronic supplementary material.

Supplementary Material 1

Supplementary Material 2

## Data Availability

The raw sequence data have been uploaded to NBCI GenBank. The BioSample accession is SAMN44272356; the BioProject accession is PRJNA1172446; and the SRA accession is PRJNA1172446. In addition, the entire complete mitogenome and plastome sequences with gene annotation have been submitted under accession PQ467906-PQ467908 and PQ492240, respectively.

## Abbreviations

LSC: Large single copy
SSC: Small single copy
IR: Inverted repeat
PCG: Protein coding gene
Kb: Killobase pair
Mb: Megabase pair
Gb: Gigabase pair
bp: Base pair
DNA: Deoxyribo nucleic acid
SSR: Simple sequence repeat
RSCU: Relative synonymous codon usage
GC: Guanine-cytosine
AT: Adenine-thymine
U: Uracil
NCBI: National Center for Biotechnology Information
Ka: Nonsynonymous substitution site
Ks: Synonymous substitution sit
