# Supplementary material for: Report on the complete organelle genomes of Orobanche Filicicola Nakai ex Hyun, Y. S. Lim & H. C. Shin (Orobanchaceae): insights from comparison with Orobanchaceae plant genomes
Source: BMC Genomics. 2025 Feb 17;26:157. doi: 10.1186/s12864-025-11298-2 (PMC11834515; doi:10.1186/s12864-025-11298-2)
Supplement: Supplementary file 1 — Supplementary Material 1 [file 12864_2025_11298_MOESM1_ESM.docx]

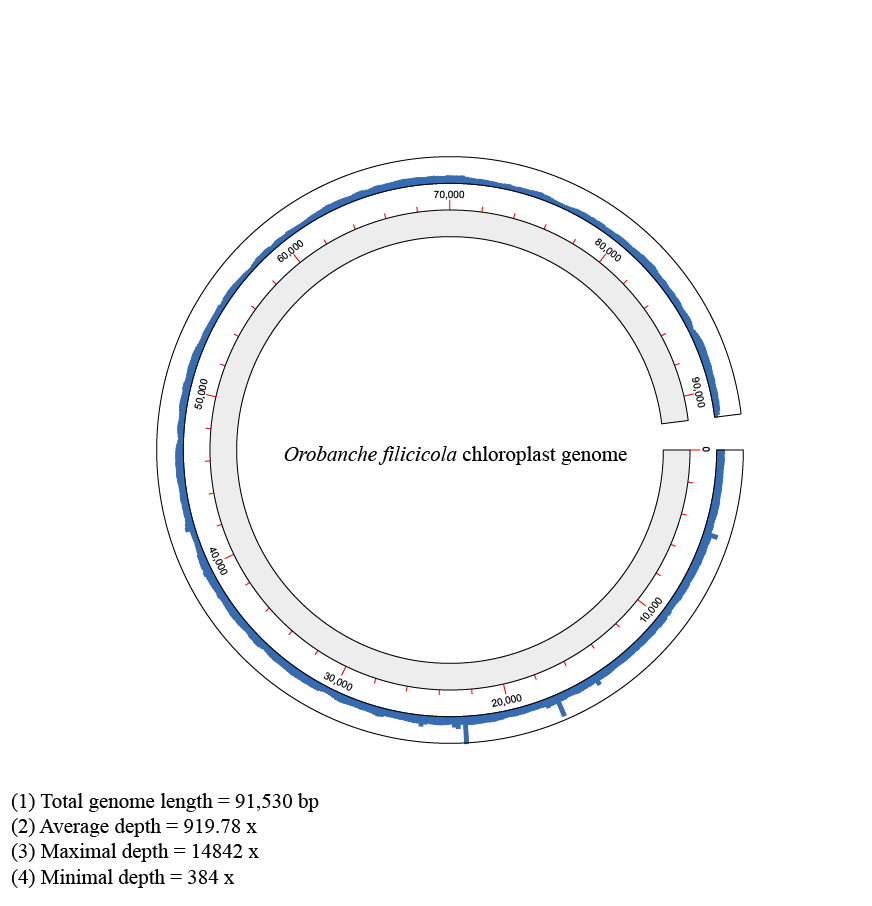


Figure S1. The sequencing depth and coverage map for Chloroplast genome of *O. filicicola*


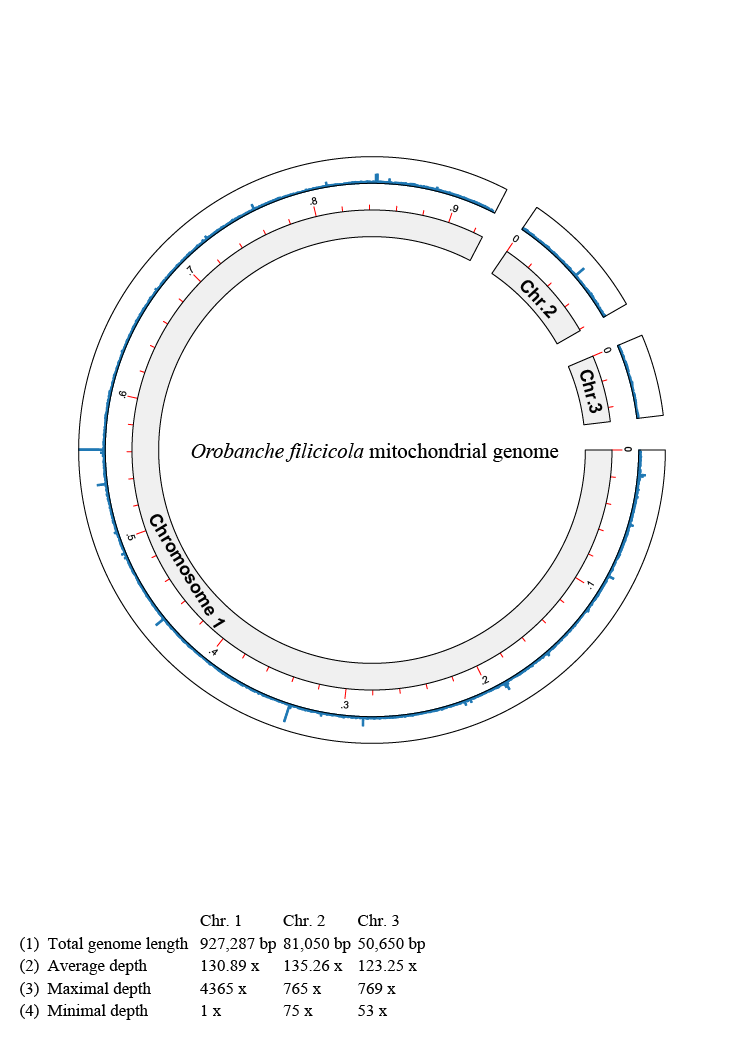


Figure S2. The sequencing depth and coverage map for mitochondrial genome of *O. filicicola*


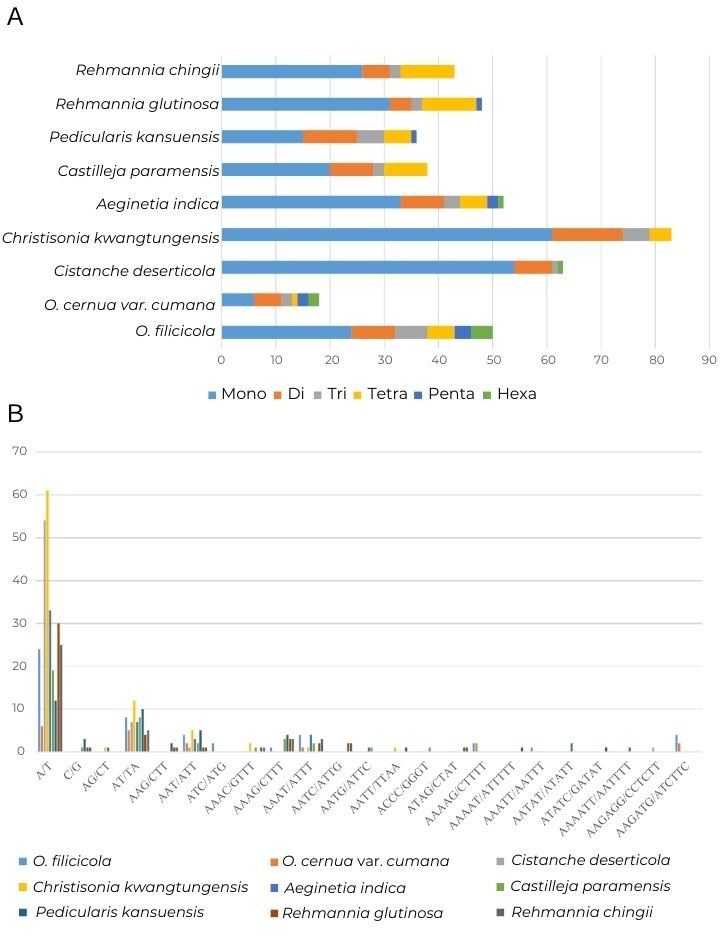


Figure S3. Analysis of simple sequence repeats (SSRs) in the chloroplast genomes of nine Orobanchaceae species. A. Frequency of different types of SSRs. B Number of six types of SSRs.


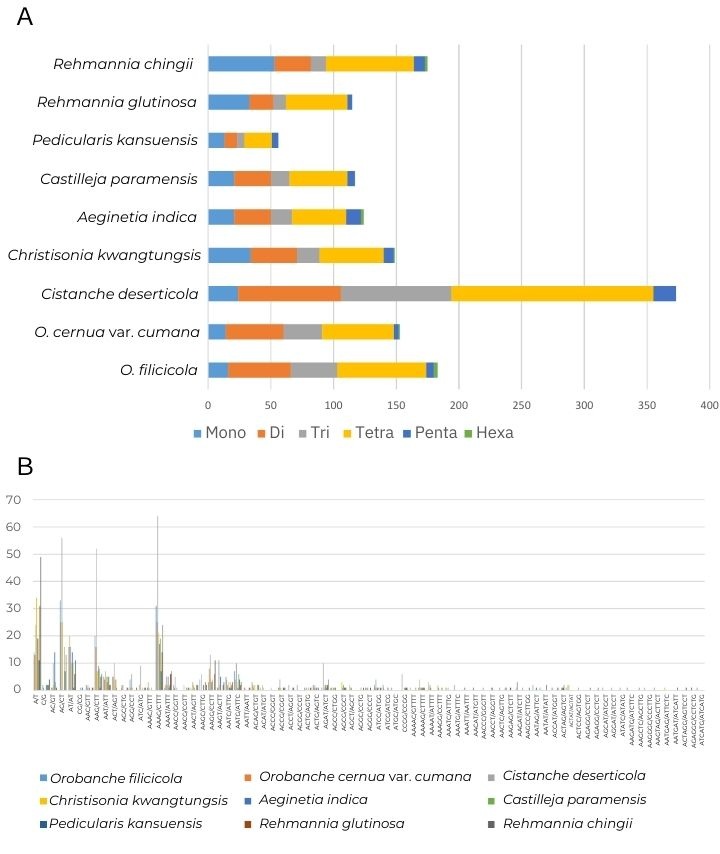


Figure S4. Analysis of simple sequence repeats (SSRs) in the mitochondria genomes of nine Orobanchaceae species. A. Frequency of different types of SSRs. B Number of six types of SSRs.


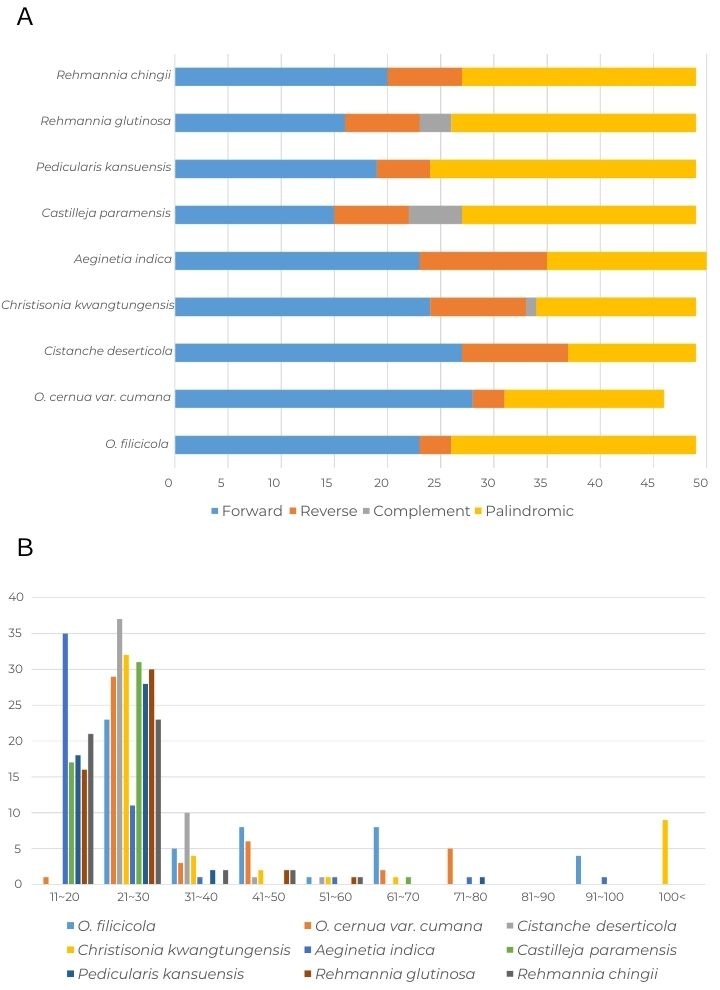


Figure S5. Comparison of long repeats in the chloroplast genomes of nine Orobanchaceae species. a. Number of different types of long repeats. b. Number of repeats of each length.


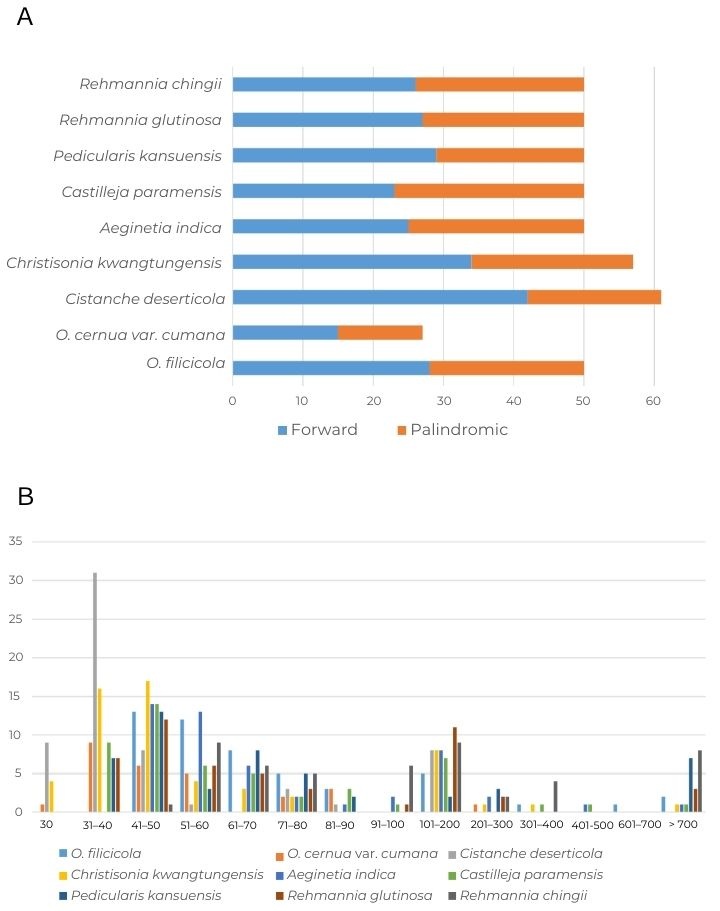


Figure S6. Comparison of long repeats in the mitochondria genomes of nine Orobanchaceae species. a. Number of different types of long repeats. b. Number of repeats of each length.
